# Supplementary material for: An Advanced Automated Patch Clamp Protocol Design to Investigate Drug—Ion Channel Binding Dynamics
Source: Front Pharmacol. 2021 Sep 28;12:738260. doi: 10.3389/fphar.2021.738260 (PMC8513526; doi:10.3389/fphar.2021.738260)
Supplement: Supplementary file 1 [file Image1.pdf]

Supplementary Material

An advanced automated patch clamp protocol design to investigate drug – ion channel binding dynamics

Peter Lukacs[1], Krisztina Pesti[2,3], Mátyás C. Földi[1,2], Katalin Zboray[1], Adam V. Toth[1,2], Gábor Papp[4], Arpad Mike[1,2]*

Supplementary Figure 1. Assessing the accuracy of automated fitting

Peak amplitude plots and fitted parameter plots in both panels A and B are identical with the data shown in Fig. 2 of the paper. Here, however, we also show *RMSE* and E_{rel} values throughout the whole experiment. Large E_{rel} values at pulses #7 and #17 are natural, because the fitting procedure minimizes absolute, not relative, squared errors, and these pulses evoke the lowest amplitude currents especially in the presence of an inhibitor. In the case of chlorpromazine and imipramine, we also see a larger relative error for pulse #6. This occurs because although the inhibition after 64 ms hyperpolarization (pulse #6) should be less than the inhibition after 32 ms hyperpolarization (pulse #12), with some of the compounds (imipramine and chlorpromazine) this was not the case (see also Fig. 3D). These compounds have slow micro-dynamics, and therefore they do not only "remember" the 64 ms hyperpolarization, but also that it was preceded by a series of prolonged depolarizations. Riluzole and trazodone, on the other hand, have fast micro-dynamics and have no "memory" of what happened several tens of milliseconds earlier.

Fig. S1

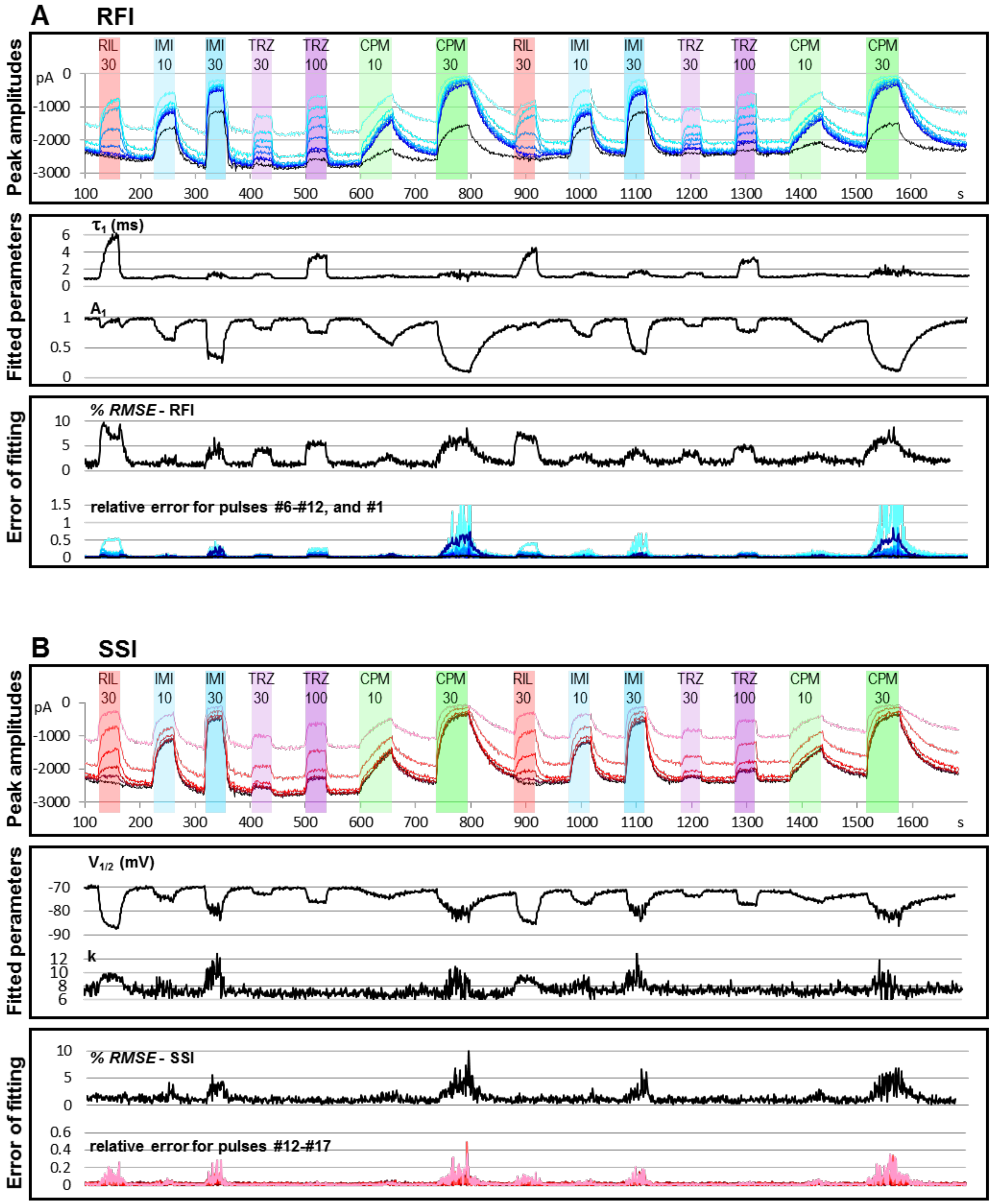
A
RFI
RIL 30
IMI 10
IMI 30
TRZ 30
TRZ 100
CPM 10
CPM 30
Peak amplitudes
pA
Fitted perameters
τ1 (ms)
A1
Error of fitting
% RMSE - RFI
relative error for pulses #6-#12, and #1
B
SSI
V1/2 (mV)
k
% RMSE - SSI
relative error for pulses #12-#17
